# Drug-Resistant Tuberculosis in Rural Eastern Cape: Integrating Whole-Genome Sequencing, Conceptual Transmission Modelling and Community-Based Participatory Research

**DOI:** 10.3390/idr18050110

**Published:** 2026-09-29

**Authors:** Ncomeka Sineke, Lindiwe Modest Faye, Ntandazo Dlatu, Saturnin Ombinda-Lemboumba, Teke Apalata

**Affiliations:** 1 WSU–TB Research Group, School of Pathology, Faculty of Medicine and Health Sciences, Walter Sisulu University, Private Bag X1, Mthatha 5100, South Africa; lfaye@wsu.ac.za (L.M.F.); tapalata@wsu.ac.za (T.A.); 2 Walter Sisulu Institute for Clinical Governance, Healthcare Administration, School of Public Health, Faculty of Medicine and Health Sciences, Walter Sisulu University, Mthatha 5099, South Africa; ndlatu@wsu.ac.za; 3 Biophotonics, Photonic Centre, Manufacturing Cluster, Council for Scientific and Industrial Research (CSIR), Pretoria 0001, South Africa; sombindalemboumba@csir.co.za

**Keywords:** Tuberculosis, drug-resistant tuberculosis, whole-genome sequencing, Community-Based Participatory Research, transmission modelling, genomic epidemiology, rural health, South Africa

## Abstract

Background: Drug-resistant tuberculosis (DR-TB) remains a major challenge in high-burden rural settings where biological, health-system, and social determinants interact. This study integrated whole-genome sequencing (WGS), mathematical modelling and Community-Based Participatory Research (CBPR)-informed principles to examine DR-TB within a conceptual biosocial framework. Methods: WGS data from 32 *Mycobacterium tuberculosis* isolates were analysed for resistance-associated mutations, phylogenetic lineages and genomic relatedness. A deterministic two-strain model explored drug-susceptible (DS) and DR-TB transmission. Hypothetical CBPR-informed scenarios modified transmission (β), treatment initiation (γ) and resistance amplification (α) parameters over 10 years. Results: Twenty-seven isolates (84.4%) showed resistance to at least one anti-TB drug, and 15 (46.9%) met MDR-TB criteria. Lineages 2 and 4 predominated. Under predefined assumptions, community-informed scenarios produced approximately 40–60% reductions in modelled DS-TB transmission and 20–35% reductions in DR-TB transmission relative to baseline. Conclusions: Integrating genomic evidence with transmission modelling and community-informed assumptions provides a useful hypothesis-generating framework for examining DR-TB. However, the modelled scenarios are exploratory and do not demonstrate the effectiveness of the CBPR intervention. Prospective studies using larger genomic datasets, longitudinal epidemiological data, and empirically measured community-level parameters are required.

## 1. Introduction

Tuberculosis (TB) remains a major global public health challenge, particularly in high-burden and resource-constrained settings [1]. Drug-resistant tuberculosis (DR-TB) further threatens TB control by reducing treatment effectiveness, increasing therapeutic complexity, and contributing to adverse outcomes [2]. In South Africa, both transmission of resistant Mycobacterium tuberculosis strains and amplification of resistance during treatment contribute to the continuing DR-TB burden [3,4]. Whole-genome sequencing (WGS) has strengthened TB molecular epidemiology by enabling high-resolution characterization of resistance-associated mutations, phylogenetic lineages, and genomic relatedness [5]. When combined with epidemiological information, genomic data can support investigation of possible transmission patterns and circulating resistant strains [6,7]. However, genomic similarity alone cannot establish direct person-to-person transmission, particularly when contact, spatial, and temporal linkage data are unavailable. Genomic evidence should therefore complement conventional epidemiological investigation [8]. Mathematical transmission models provide an additional approach to examining processes that influence drug-susceptible (DS) and DR-TB dynamics and to exploring hypothetical intervention scenarios [9]. Their outputs, however, depend on model structure, parameterization, and assumptions and should not be interpreted as observed population effects unless appropriately calibrated and validated. Importantly, DR-TB transmission is influenced not only by pathogen biology but also by behavioural, social, structural, and health-system conditions. Delayed healthcare seeking, treatment interruption, stigma, poverty, geographical barriers, and limited access to diagnosis and treatment may contribute to ongoing transmission and poor treatment continuity [10]. These challenges are particularly relevant in rural South African settings. Community-Based Participatory Research (CBPR) provides a framework for incorporating community perspectives into the identification of health priorities, the development of interventions, and their evaluation [11]. Community-engaged approaches may influence TB transmission by promoting earlier healthcare-seeking, improving treatment continuity, strengthening infection-prevention practices, enhancing health literacy, and strengthening trust in healthcare services. However, these effects require empirical evaluation. Few studies have explicitly integrated community-informed mechanisms with genomic evidence and mathematical modelling of DR-TB transmission.

This study, therefore, integrated WGS, exploratory mathematical transmission modelling, and CBPR-informed scenario development within a conceptual biosocial framework. The objectives were to characterize resistance-associated mutations, phylogenetic lineages, and genomic relatedness; examine DS-TB and DR-TB transmission dynamics using an exploratory two-strain model; and assess how predefined community-informed changes in transmission, treatment initiation, and resistance-amplification parameters could alter projected transmission dynamics. The modeling scenarios were hypothesis-generating and were not designed to estimate the effectiveness of prospectively implemented CBPR interventions.

## 2. Methodology

### 2.1. Study Design and Setting

This retrospective laboratory-based study integrated whole-genome sequencing (WGS) with deterministic mathematical transmission modelling to examine drug-sensitive (DS) and drug-resistant (DR) tuberculosis (TB). WGS was used to characterise resistance-associated mutations, phylogenetic lineages, and genomic relatedness, while mathematical modelling explored hypothetical DS-TB and DR-TB transmission scenarios. Community-Based Participatory Research (CBPR) was not conducted or prospectively evaluated as an intervention in this study. Rather, CBPR principles were used solely as a conceptual framework to inform predefined hypothetical modifications of selected epidemiological parameters. Accordingly, empirical genomic findings were clearly distinguished from model-based projections and conceptual community-informed assumptions.

**Figure 1:**
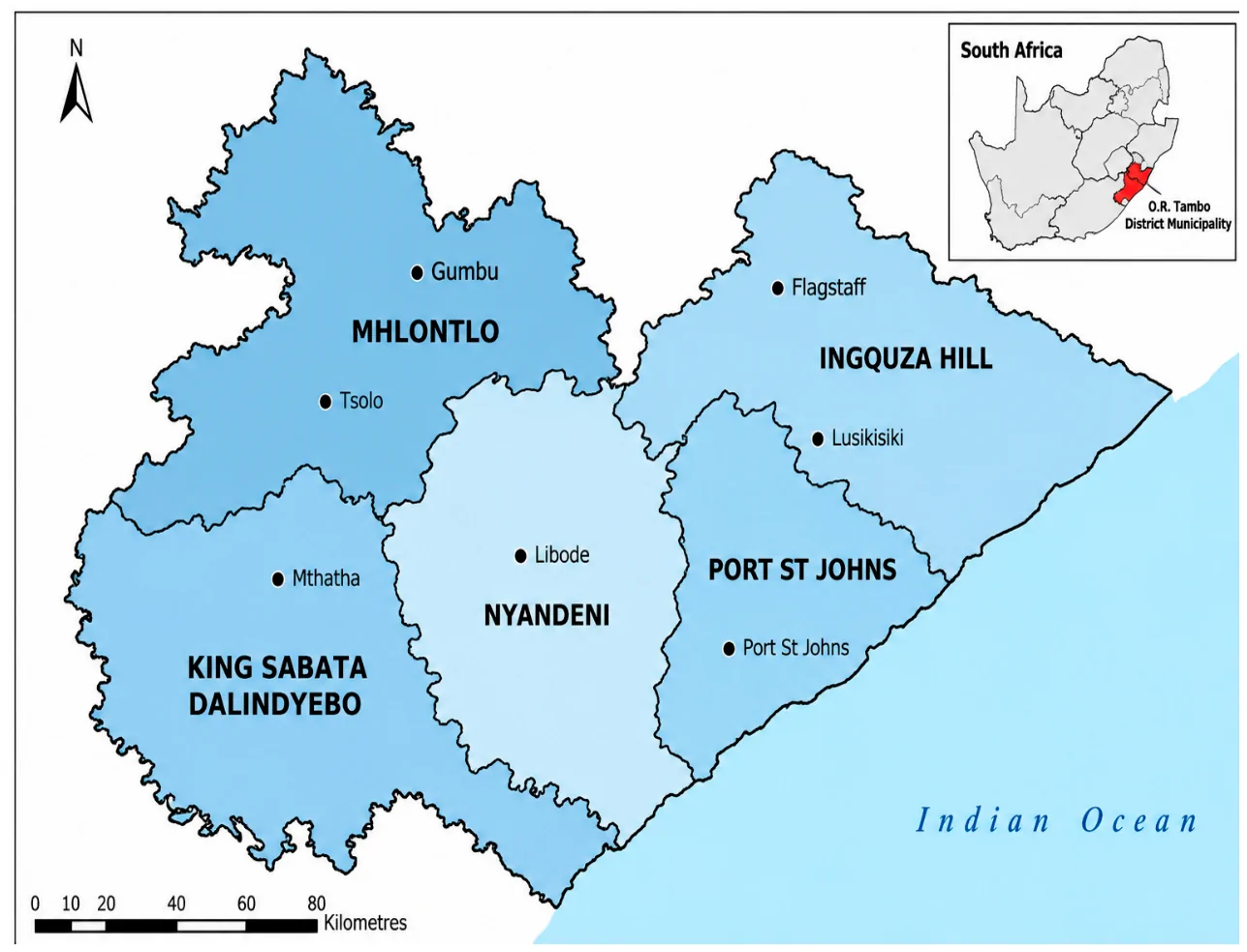
O.R. Tambo District Municipality administrative boundaries. Adapted from Municipalities of South Africa [13].

The study was conducted in O.R. Tambo District Municipality, Eastern Cape Province, South Africa (Figure 1). The predominantly rural district comprises five local municipalities and has a population exceeding 1.5 million, with approximately 93% of residents living in rural communities [12,13,14,15]. Culture-confirmed *Mycobacterium tuberculosis* isolates were obtained from patients receiving routine TB diagnostic and treatment services at public healthcare facilities between 2018 and 2020. Laboratory confirmation, culture, and storage were undertaken through the National Health Laboratory Service. The 32 isolates included in the genomic analysis constituted a selected retrospective sample of archived, culture-confirmed *M. tuberculosis* isolates rather than a population-representative sample of all notified TB cases in O.R. Tambo District. Consequently, the proportion of MDR-TB observed among the sequenced isolates (46.9%) should not be interpreted as the population prevalence or notification proportion of MDR-TB in the district. Population-level surveillance indicators and sample-specific genomic resistance proportions represent distinct epidemiological measures and were therefore interpreted separately.

### 2.2. Case Ascertainment and Whole-Genome Sequencing

Archived culture-confirmed *Mycobacterium tuberculosis* isolates were selected based on culture viability, availability of laboratory records, DNA quality, and successful sequencing. Contaminated or non-viable cultures, isolates with inadequate DNA, sequencing quality-control failures, duplicates, and isolates with incomplete genomic data were excluded. No prospective patient recruitment was undertaken.

Of 36 isolates processed, 32 (88.9%) successfully underwent whole-genome sequencing (WGS). DNA was fragmented using the NEB Ultra II FS kit, size-selected using AMPure XP beads, ligated to Illumina adapters, indexed, normalised to 4 nM, and sequenced using the Illumina MiSeq v3 600-cycle kit (Illumina, Inc., San Diego, CA, USA), generating paired-end 2 × 300 bp reads.

Resistance profiling and lineage classification were performed with TB-Profiler against the M. tuberculosis H37Rv reference genome, followed by additional processing with the Unified Sequence Analysis Pipeline. Reads were quality-trimmed using Trimmomatic and aligned to H37Rv (NC000962.3) using BWA, SMALT, and Novoalign. Variants were identified using SAMtools and GATK. Resistance-associated loci included *rpoB*, *katG*, the *fabG1/inhA* promoter region, *gyrA*, and *rrs*.

MDR-TB was defined as resistance to at least rifampicin and isoniazid; pre-XDR-TB as MDR/RR-TB with additional resistance to any fluoroquinolone; and XDR-TB as MDR/RR-TB with fluoroquinolone resistance and additional resistance to bedaquiline or linezolid. *rrs* mutations were not used to assign pre-XDR-TB or XDR-TB status. Pairwise SNP distances were not used to define transmission clusters; genomic similarities were therefore interpreted as genetic relatedness rather than confirmed transmission.

### 2.3. Mathematical Transmission Model and Parameterisation

A deterministic two-strain compartmental model was developed to explore the transmission dynamics of drug-sensitive (DS) and drug-resistant (DR) tuberculosis and to examine the potential effects of community-informed interventions on these dynamics. The model was intended as an exploratory, hypothesis-generating framework rather than a calibrated forecasting model for the O.R. Tambo District. Mathematical modelling approaches incorporating treatment and control interventions have previously been applied to investigate TB transmission dynamics [9].

#### 2.3.1. Model Structure

The total model population, *N*, was divided into nine epidemiological compartments: susceptible individuals (S); individuals latently infected with DS-TB (E_s_); individuals with infectious DS-TB (I_s_); individuals receiving treatment for DS-TB (T_s_); individuals recovered from DS-TB (R_s_); individuals latently infected with DR-TB (Eᵣ); individuals with infectious DR-TB (Iᵣ); individuals receiving treatment for DR-TB (Tᵣ); and individuals recovered from DR-TB (Rᵣ).

The total population was therefore defined as

*N*
= S + E_s_ + I_s_ + T_s_ + R_s_ + Eᵣ + Iᵣ + Tᵣ + Rᵣ

Separate transmission pathways were specified for DS-TB and DR-TB. Susceptible individuals acquired DS-TB or DR-TB following effective contact with infectious individuals. The respective forces of infection were defined as

λ_s_ = β_s_I_s_/*N*

λᵣ = βᵣIᵣ/*N*

where β_s_ and βᵣ denote the effective transmission coefficients for DS-TB and DR-TB, respectively.

Individuals with latent infection progressed to active infectious disease at rates σ_s_ and σᵣ. Infectious individuals initiated treatment at rates γ_s_ and γᵣ, while treated individuals recovered at rates ρ_s_ and ρᵣ. Background mortality was represented by μ, and additional TB-related mortality was represented by d_s_ and dᵣ for DS-TB and DR-TB, respectively.

Resistance amplification during DS-TB treatment was represented by the parameter α. This pathway was included to distinguish primary transmission of an already resistant strain, represented by λᵣ, from the amplification of resistance during treatment, represented by α.

The resulting system of ordinary differential equations was

dS/dt = Λ − (λ_s_ + λᵣ)S − μS

dE_s_/dt = λ_s_S − (σ_s_ + μ)E_s_

dI_s_/dt = σ_s_E_s_ − (γ_s_ + μ + d_s_)I_s_

dT_s_/dt = γ_s_I_s_ − (ρ_s_ + α + μ)T_s_

dR_s_/dt = ρ_s_T_s_ − μR_s_

dEᵣ/dt = λᵣS + αT_s_ − (σᵣ + μ)Eᵣ

dIᵣ/dt = σᵣEᵣ − (γᵣ + μ + dᵣ)Iᵣ

dTᵣ/dt = γᵣIᵣ − (ρᵣ + μ)Tᵣ

dRᵣ/dt = ρᵣTᵣ − μRᵣ

Here, Λ represents recruitment into the susceptible population.

#### 2.3.2. Model Assumptions

The model assumed homogeneous mixing, constant demographic conditions over the simulation period, and a closed model population apart from demographic recruitment and mortality. Reinfection, migration, age structure, HIV co-infection, heterogeneous contact patterns, and stochastic variation were not explicitly represented. These simplifications were considered appropriate for the exploratory purpose of comparing the relative behaviour of DS-TB and DR-TB under predefined intervention scenarios rather than reproducing the complete epidemiology of TB in the study population.

Importantly, the 32 WGS isolates were not used to estimate the dynamic epidemiological parameters of the model. Instead, the genomic findings informed its biological structure by demonstrating the coexistence of drug-sensitive and drug-resistant profiles and identifying resistance-associated mutations consistent with the need to represent separate DS-TB and DR-TB pathways and resistance amplification. Consequently, observed mutation frequencies, resistance-class frequencies, lineage frequencies, and genomic relatedness were not treated as direct estimates of β, σ, γ, ρ, α, or R_0_.

#### 2.3.3. Model Parameterisation

The complete parameter set used for the simulations is presented in Table 1. Baseline parameter values were informed by the published TB epidemiological and mathematical-modelling literature where suitable estimates were available and by explicit modelling assumptions where empirical estimates were unavailable. Mathematical TB models incorporating treatment, incomplete treatment, first- and second-line treatment, heterogeneous disease progression, and intervention strategies provided the conceptual basis for parameter selection [9].

The baseline effective transmission coefficients were set at β_s_ = 0.44 year^−1^ for DS-TB and βᵣ = 1.10 year^−1^ for DR-TB. Progression from latent infection to active disease was represented by σ_s_ = σᵣ = 0.10 year^−1^. Baseline treatment-initiation rates were γ_s_ = 6.50 year^−1^ for DS-TB and γᵣ = 4.00 year^−1^ for DR-TB. The lower treatment-initiation rate specified for DR-TB represented a longer pathway to initiation of an appropriate resistant-TB regimen.

Successful treatment recovery rates were set at ρ_s_ = 0.85 year^−1^ and ρᵣ = 0.60 year^−1^. Resistance amplification during DS-TB treatment was represented by α = 0.10 year^−1^. Background mortality was set at μ = 0.011 year^−1^, while additional TB-related mortality rates were d_s_ = 0.05 year^−1^ for DS-TB and dᵣ = 0.10 year^−1^ for DR-TB. Simulations were conducted for an initial hypothetical model population of 100,000 individuals over a 10-year time horizon.

Table 1 summarises the epidemiological parameters used to parameterise the deterministic two-strain model under baseline, moderate community education, and high-intensity community engagement scenarios. Parameters describing biological progression, recovery, background mortality, and TB-related mortality were held constant across the intervention scenarios. Intervention effects were introduced specifically through parameters considered plausibly responsive to improved health literacy, care-seeking, treatment continuity, and community participation.

The numerical parameterisation of the DS–DR transmission model and the corresponding baseline, moderate community education and high-intensity community engagement scenarios are presented in Table 1. To improve transparency regarding the origin and interpretation of these parameters, Table 2 provides the supporting epidemiological evidence and modelling justification for each parameter. This distinction was particularly important because the WGS component of the study was not designed to estimate population-level transmission, progression, treatment or mortality rates. Consequently, the genomic data informed the biological structure of the model and the representation of DS-TB and DR-TB pathways but were not used to derive numerical estimates of *β*, *σ*, *γ*, *ρ*, *α*, *μ*, or *d*.

As shown in Table 2, parameters describing TB natural history, treatment pathways and mortality were informed by published epidemiological evidence where appropriate [19,20,21,22,23,24,27,29]. In contrast, parameters for which direct study-specific estimates were unavailable were explicitly treated as modelling assumptions. In particular, the DS-TB and DR-TB transmission coefficients (*β_s_* and *β_r_*) were model-calibrated parameters used to generate comparative transmission scenarios rather than estimates obtained from the 32 sequenced isolates [16,17,18]. Similarly, the resistance-amplification parameter (*α*) was introduced to represent the potential development or amplification of resistance during treatment, while reductions in *α* under the intervention scenarios represented the hypothesised effect of improved adherence and retention in care [25,26].

Table 1 and Table 2 therefore separate numerical model inputs from their evidence base and assumptions. This approach allows the simulated outcomes to be interpreted as conditional, hypothesis-generating projections rather than empirical forecasts for the study district. The moderate and high-intensity intervention scenarios should likewise be interpreted as predefined counterfactual scenarios designed to examine how progressively reduced transmission and resistance amplification, together with earlier treatment initiation, could alter DS-TB and DR-TB dynamics.

#### 2.3.4. Community-Informed Intervention Scenarios

Three scenarios were examined: baseline, moderate community education, and high-intensity community engagement.

The baseline scenario represented model behaviour in the absence of an additional community-engaged health-literacy intervention.

Under the moderate community education scenario, the effective transmission coefficients for DS-TB and DR-TB were reduced by 25%, treatment-initiation rates were increased by 25%, and resistance amplification during DS-TB treatment was reduced by 50%. Accordingly, β_s_ decreased from 0.44 to 0.33 year^−1^ and βᵣ from 1.10 to 0.83 year^−1^, γ_s_ increased from 6.50 to 8.13 year^−1^ and γᵣ from 4.00 to 5.00 year^−1^, and α decreased from 0.10 to 0.05 year^−1^.

Under the high-intensity community engagement scenario, the effective transmission coefficients were reduced by 50%, treatment-initiation rates were increased by 50%, and resistance amplification was reduced by 80%. Thus, β_s_ decreased to 0.22 year^−1^ and βᵣ to 0.55 year^−1^, γ_s_ increased to 9.75 year^−1^ and γᵣ to 6.00 year^−1^, and α decreased to 0.02 year^−1^.

These modifications were intended to represent plausible pathways through which community-engaged TB health literacy could influence transmission. Reduced β represents improved infection-prevention behaviour, including earlier symptom recognition, ventilation, cough hygiene, and reduced exposure to infectious individuals. Increased γ represents earlier healthcare-seeking, diagnosis, linkage to care, and treatment initiation. Reduced α represents improved treatment adherence and continuity, thereby decreasing opportunities for resistance amplification.

The moderate and high-intensity parameter modifications were predefined counterfactual modelling assumptions. They were not estimated from the WGS dataset, prospectively implemented community interventions, or empirical CBPR effectiveness data. The scenarios were therefore designed to examine the direction and magnitude of model responses to progressively stronger community-informed changes rather than to estimate the effectiveness of a specific intervention.

#### 2.3.5. Reproduction Numbers

The basic reproduction numbers for DS-TB and DR-TB were derived from the corresponding infection subsystems using the next-generation matrix approach. In simplified form, transmission potential depends on the transmission coefficient, probability of progression to infectious disease, and expected duration of infectiousness. For each strain, this relationship can be represented as

R_0s_ = β_s_σ_s_/[(σ_s_ + μ)(γ_s_ + μ + d_s_)]

R_0_ᵣ = βᵣσᵣ/[(σᵣ + μ)(γᵣ + μ + dᵣ)]

The reproduction numbers were therefore derived model quantities rather than parameters estimated from the 32 WGS isolates.

Under the selected baseline parameterisation, the model produced illustrative reproduction numbers of approximately 1.29 for DS-TB and 2.04 for DR-TB. Under the moderate community-education scenario, the corresponding values were approximately 0.97 and 1.53, respectively. Under the high-intensity scenario, the corresponding model values were approximately 0.65 for DS-TB and 1.02 for DR-TB. These values represent deterministic scenario outputs conditional on the specified parameterisation and should not be interpreted as empirical reproduction-number estimates for the O.R. Tambo District.

#### 2.3.6. Simulation and Sensitivity Analysis

The system of ordinary differential equations was simulated over a 10-year time horizon under each intervention scenario. The primary model outputs were the trajectories of active DS-TB and DR-TB, the relative contribution of resistant disease over time, and the strain-specific reproduction numbers.

Because the model was exploratory and was not fitted to longitudinal incidence, prevalence, notification, or contact-tracing data, formal statistical confidence or Bayesian credible intervals were not calculated. Instead, uncertainty was examined through deterministic scenario and sensitivity analyses. Key parameters governing transmission and intervention effects, particularly β_s_, βᵣ, γ_s_, γᵣ, and α, were varied around their baseline values to evaluate the sensitivity of the reproduction numbers and projected transmission trajectories to alternative assumptions.

Particular attention was given to the transmission coefficients because changes in β_s_ and βᵣ directly alter the forces of infection. Treatment-initiation rates were varied to examine the consequences of shortening or extending the infectious period, while α was varied to evaluate the influence of resistance amplification during DS-TB treatment. The moderate and high-intensity community scenarios themselves therefore also functioned as structured sensitivity analyses of progressively stronger reductions in transmission and resistance amplification and increases in treatment initiation.

Model outputs were interpreted as conditional projections rather than forecasts. No attempt was made to infer district-specific transmission rates, predict future case numbers, or estimate the observed effectiveness of community engagement from the simulations. Instead, the model was used to examine whether biologically and socially plausible changes in transmission, treatment initiation, and resistance amplification could alter the relative dynamics of DS-TB and DR-TB and to generate hypotheses for future empirical investigation.

Generative artificial intelligence (GenAI) was used during manuscript preparation to assist with language refinement, organisation and presentation of manuscript text, and refinement of the conceptual presentation of the mathematical modelling framework. ChatGPT (OpenAI, San Francisco, CA, USA), powered by GPT-5.6 Sol, was used for these purposes. The GenAI tool was not used to generate the whole-genome sequencing data, perform laboratory procedures, determine resistance classifications, or independently generate or validate the study findings. All AI-assisted outputs were critically reviewed, verified and revised by the authors against the underlying study data and relevant literature. The authors take full responsibility for the accuracy, interpretation and final content of the manuscript.

### 2.4. Community-Informed Conceptual Scenarios and Simulation

Community-Based Participatory Research (CBPR) was not conducted, prospectively implemented, or evaluated as an intervention in the present study, and no primary community-level data were collected for this analysis. Instead, CBPR principles were used solely as a conceptual framework for constructing hypothetical transmission-model scenarios representing plausible pathways through which community-oriented approaches could influence TB transmission and care.

Three model parameters were modified under these predefined scenarios: transmission coefficients (β_s_ and βᵣ), treatment-initiation rates (γ_s_ and γᵣ), and the resistance-amplification parameter (α). Reductions in β_s_ and βᵣ represented hypothetical improvements in infection-prevention practices; increases in γ_s_ and γᵣ represented earlier healthcare seeking and treatment initiation; and reductions in α represented improved treatment adherence and continuity. These parameter modifications were modelling assumptions and were not derived from empirical CBPR findings, community engagement activities, or observed intervention effects.

Three scenarios were evaluated: baseline, moderate community-informed, and high-intensity community-informed. Simulations were conducted over 10 years using deterministic numerical solutions. Outcomes included projected DS-TB and DR-TB trajectories, as well as illustrative basic reproduction numbers (R_0_). Because the model was exploratory and was not calibrated to observed district-level epidemiological data, the resulting outputs were interpreted as hypothesis-generating scenario projections rather than empirical estimates, forecasts, or evidence of CBPR effectiveness. Statistical confidence or credible intervals were not calculated.

## 3. Results

### 3.1. Distribution of Drug-Sensitive and Drug-Resistant Strains

Whole-genome sequencing of 32 *Mycobacterium tuberculosis* isolates identified resistance-associated mutations to at least one anti-tuberculosis drug in 27 isolates (84.4%). In contrast, five (15.6%) had no identified resistance-associated mutations for the drugs evaluated. MDR-TB was the most common resistance category, accounting for 15 isolates (46.9%), while six isolates (18.8%) had other resistance profiles that did not meet MDR-TB criteria. Using current WHO definitions, pre-XDR-TB was assigned only where MDR/RR-TB occurred with additional fluoroquinolone resistance. The *rrs* mutations were not used to classify pre-XDR-TB or XDR-TB. XDR-TB was not assigned because sufficient validated genomic evidence of resistance to bedaquiline or linezolid was unavailable. Given the cross-sectional design, the observed resistance profiles were not interpreted as evidence of sequential acquisition or progression of resistance.

### 3.2. Lineage Distribution and Resistance Profiles

Lineage 4 (Euro-American) accounted for 46.9% (n = 15) of isolates and Lineage 2 (East Asian, including Beijing-family strains) for 40.6% (n = 13). Lineages 1 and 3 were each represented by a single isolate, while a third isolate exhibited mixed-lineage characteristics. MDR-TB occurred among both Lineage 2 and Lineage 4 isolates, whereas fluoroquinolone resistance-associated mutations were observed predominantly among Lineage 2 isolates. When fluoroquinolone resistance co-occurred with MDR/RR-TB, isolates were evaluated for pre-XDR-TB classification. Although complex resistance profiles were more frequent among Lineage 2 isolates, the small numbers within individual categories precluded statistical inference about associations between lineage and advanced drug resistance.

### 3.3. Resistance-Associated Mutations

Canonical resistance-associated mutations were identified across first- and second-line anti-tuberculosis drug targets. Rifampicin resistance-associated *rpoB* mutations were detected in 26 isolates, with *rpoB* Ser450Leu being the most frequent variant. Isoniazid resistance-associated mutations were identified in 23 isolates, predominantly the *katG* Ser315Thr and *fabG1/inhA* promoter −15C>T mutations. Fluoroquinolone resistance-associated mutations were detected in five isolates, predominantly involving *gyrA* codon 94 substitutions. The *rrs* 1401A>G mutation was identified in eight isolates. It was reported according to its established drug-resistance association rather than used to classify isolates as pre-XDR-TB or XDR-TB. Several isolates shared combinations of resistance-associated mutations; however, these similarities were not interpreted as evidence of direct transmission.

### 3.4. Genetic Relatedness of Drug-Resistant Isolates

**Figure 2:**
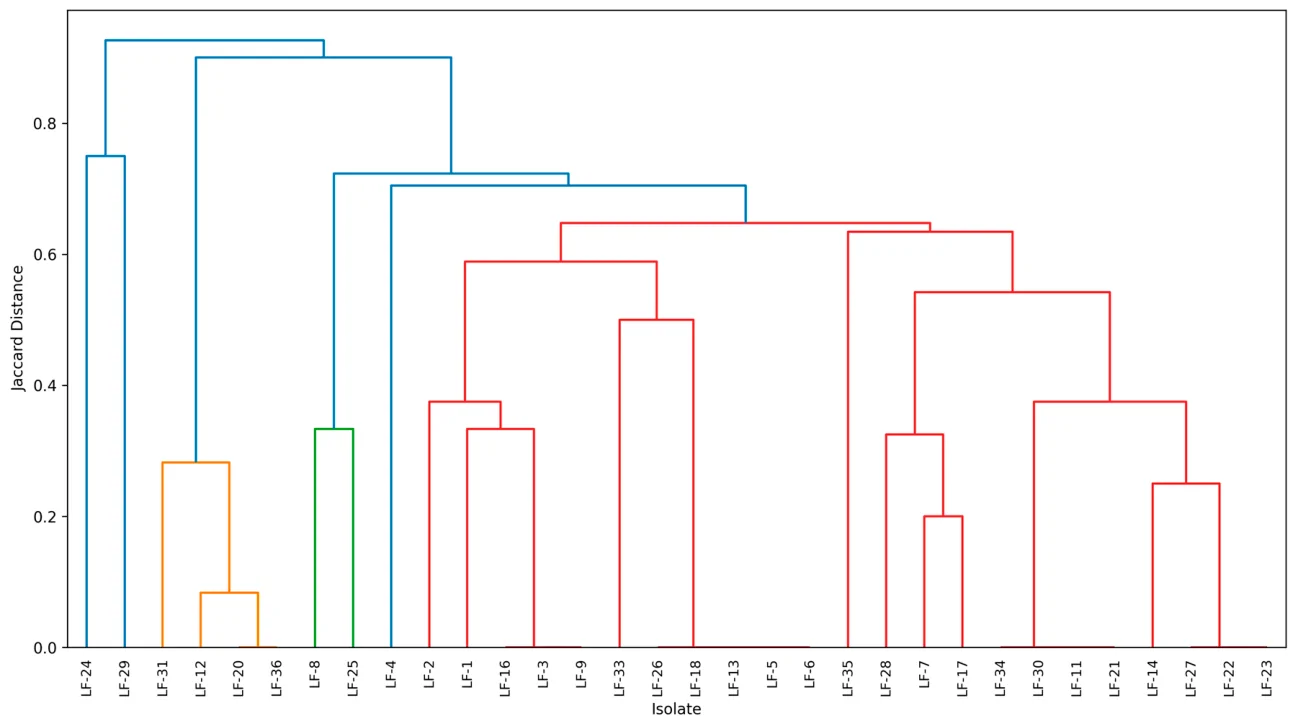
Hierarchical clustering dendrogram of whole-genome sequencing resistance mutation profiles among *Mycobacterium tuberculosis* isolates.

Comparative genomic analysis identified recurrent resistance-mutation profiles among the 32 isolates, particularly combinations involving *rpoB* Ser450Leu with *katG* Ser315Thr or the *fabG1/inhA* promoter −15C>T mutation. Hierarchical clustering of resistance profiles demonstrated groups of isolates with similar genomic resistance signatures (Figure 2). These groupings represent similarity in resistance-associated mutation profiles rather than confirmed transmission clusters. Pairwise SNP thresholds, detailed contact-tracing information, temporal relationships, and geographical linkage data were unavailable; therefore, direct person-to-person transmission or transmission-chain reconstruction could not be established. The observed patterns instead indicate genomic relatedness and recurring resistance signatures that may warrant further epidemiological investigation.

### 3.5. Mathematical Model Framework for DS–DR TB Transmission

**Figure 3:**
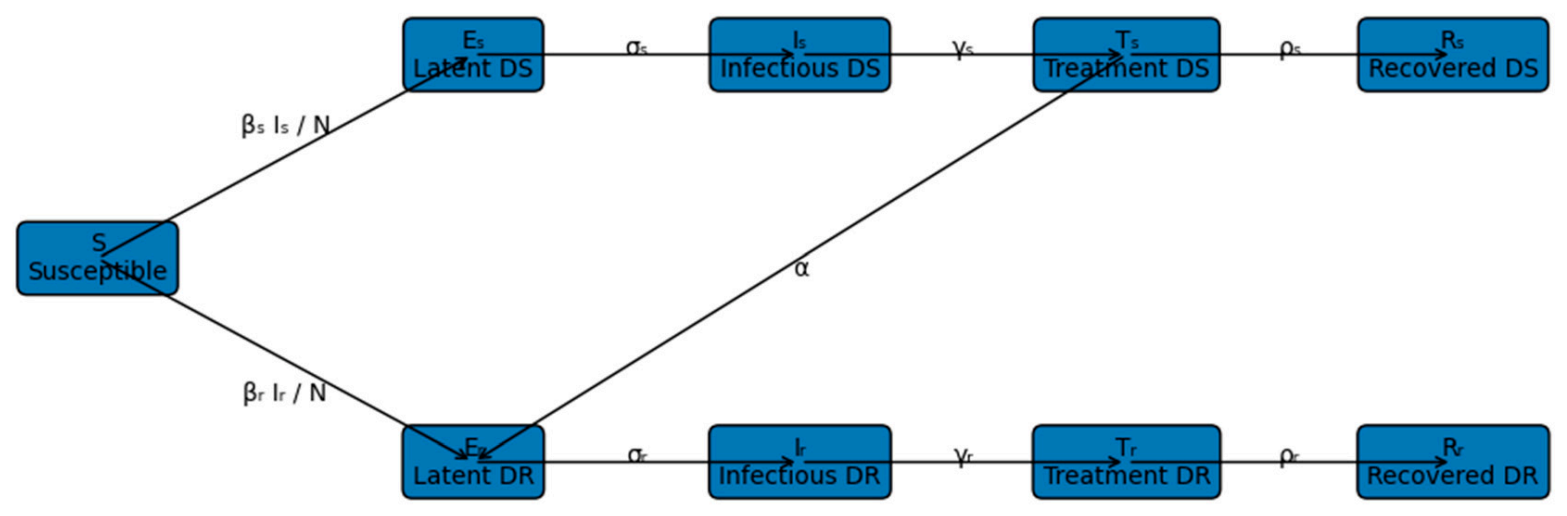
Two-strain compartmental transmission model for drug-sensitive and drug-resistant tuberculosis.

A conceptual two-strain compartmental model was used to explore the transmission dynamics of drug-sensitive (DS) and drug-resistant (DR) tuberculosis (Figure 3). The population was divided into susceptible (S), latent infection (E), infectious disease (I), treatment (T), and recovered (R) compartments, with separate states for DS-TB and DR-TB. The total population was defined as

*N*
= S + E_s_ + I_s_ + T_s_ + R_s_ + Eᵣ + Iᵣ + Tᵣ + Rᵣ

Transmission was governed by strain-specific coefficients (β_s_ and βᵣ), while progression from latent to active disease occurred at rates σ_s_ and σᵣ. Treatment initiation was represented by γ_s_ and γᵣ and recovery by ρ_s_ and ρᵣ. Drug resistance could arise from direct transmission of resistant strains or from resistance amplification during DS-TB treatment, as represented by α.

Conceptual model showing susceptible, latent, infectious, treatment, and recovered compartments for DS-TB and DR-TB. Drug resistance may arise through direct transmission of resistant strains or amplification during treatment of DS-TB.

### 3.6. Integrated Genomics-to-Transmission Framework

**Figure 4:**
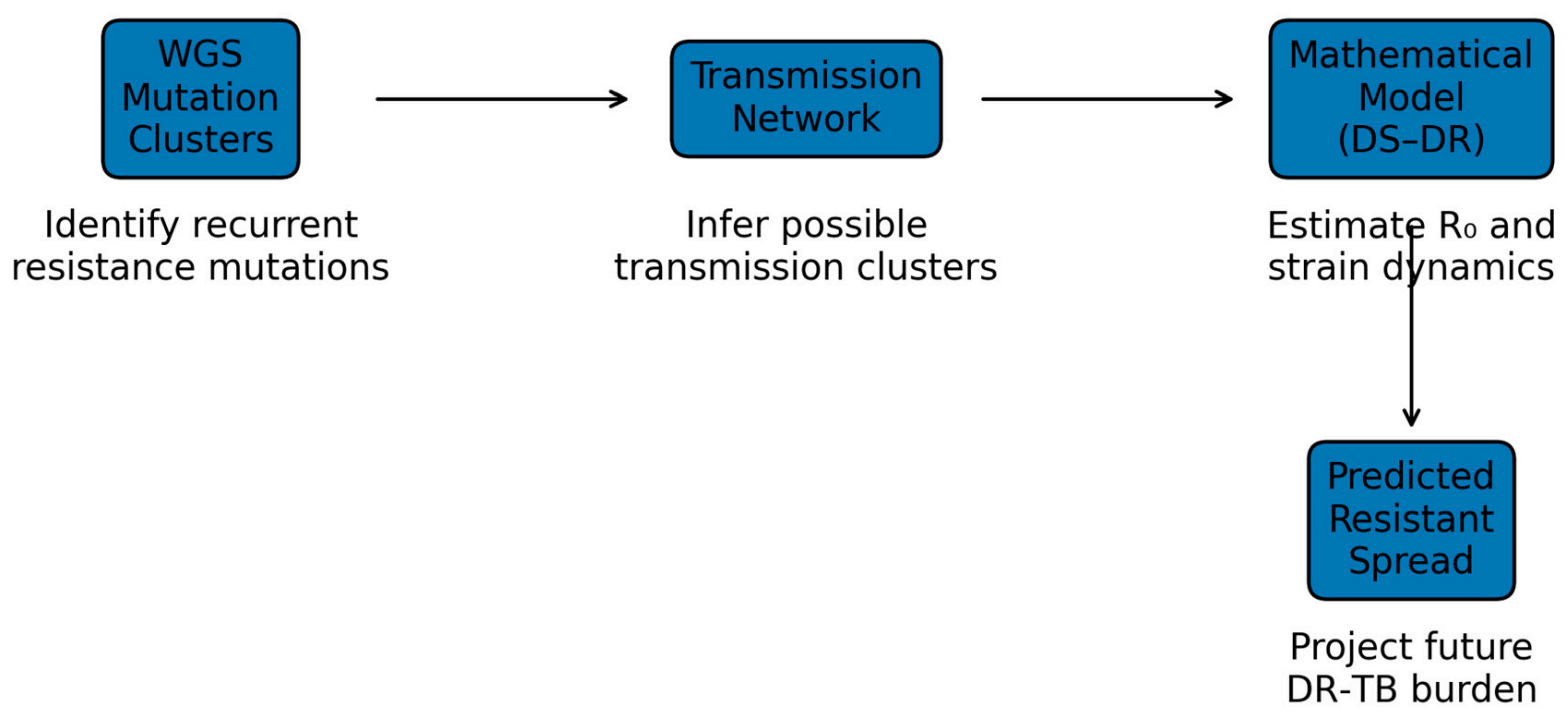
Integrated genomics-to-transmission framework for drug-resistant tuberculosis.

The integrated framework linked WGS-derived resistance profiles with the conceptual DS–DR transmission model (Figure 4). WGS identified resistance-associated mutations, lineage distributions, and recurrent resistance signatures, while the mathematical model represented their potential epidemiological context at the population level. Genomic findings informed the model’s biological structure but were not used to estimate transmission coefficients or other dynamic epidemiological parameters.

This conceptual framework illustrates the relationship between WGS-derived resistance information and mathematical transmission modelling. Genomic findings provide a biological context for the DS–DR model but do not constitute direct estimates of transmission parameters or validated transmission pathways.

### 3.7. Conceptual Resistance Pathway

**Figure 5:**
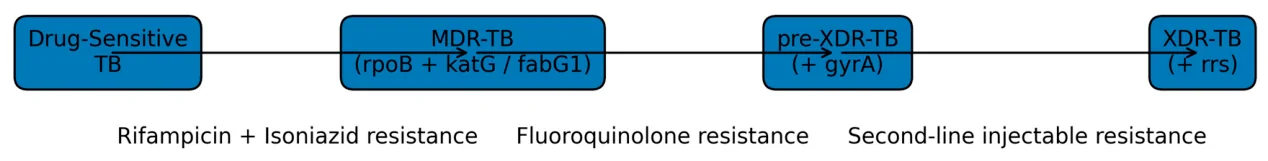
Conceptual pathway of drug resistance in *Mycobacterium tuberculosis*.

Resistance-associated mutations observed in the genomic dataset were mapped onto a conceptual resistance pathway (Figure 5). Rifampicin and isoniazid resistance were primarily associated with mutations in rpoB and katG, or in the *fabG1/inhA* promoter region, respectively. Additional fluoroquinolone resistance was associated predominantly with *gyrA* mutations. The pathway is conceptual and does not represent the longitudinal evolution of resistance observed among individual patients. In particular, the cross-sectional genomic data cannot establish sequential progression from DS-TB to MDR-TB and subsequently to more advanced resistance categories.

This conceptual representation shows resistance-associated mutations identified across major anti-tuberculosis drug classes. The pathway illustrates potential accumulation of resistance and does not represent observed longitudinal evolutionary trajectories in individual patients.

### 3.8. Baseline Model Outputs

**Figure 6:**
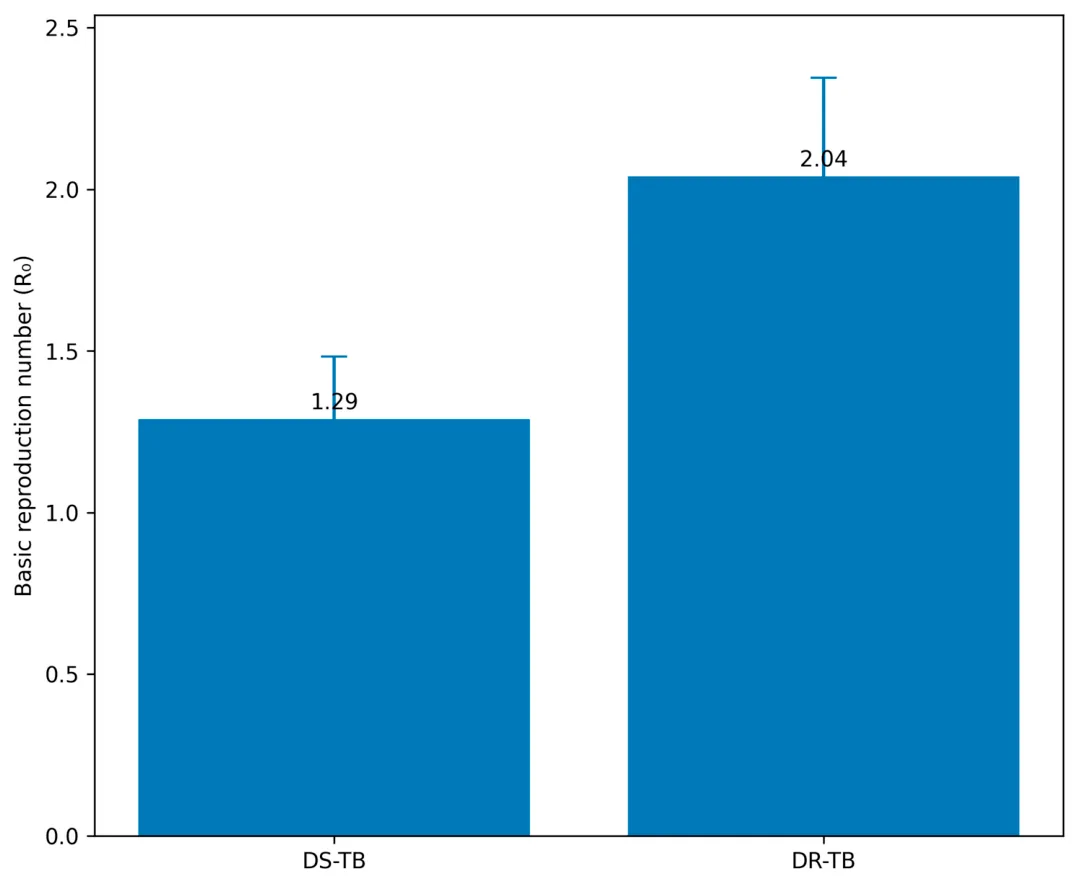
Illustrative basic reproduction number values for drug-sensitive and drug-resistant tuberculosis under the baseline scenario. Model-derived R_0_ values for DS-TB and DR-TB were generated under the selected baseline assumptions. Values represent deterministic scenario outputs rather than empirically estimated reproduction numbers for the study population.

Under the selected baseline parameter assumptions, the deterministic model produced illustrative basic reproduction number (R_0_) values of approximately 1.29 for DS-TB and 2.04 for DR-TB (Figure 6). These values represent model behaviour under specified assumptions and are not empirical estimates for the study population. Because model parameters were literature-informed rather than estimated from longitudinal study data and the model was not formally calibrated, statistical confidence or credible intervals were not calculated.

**Figure 7:**
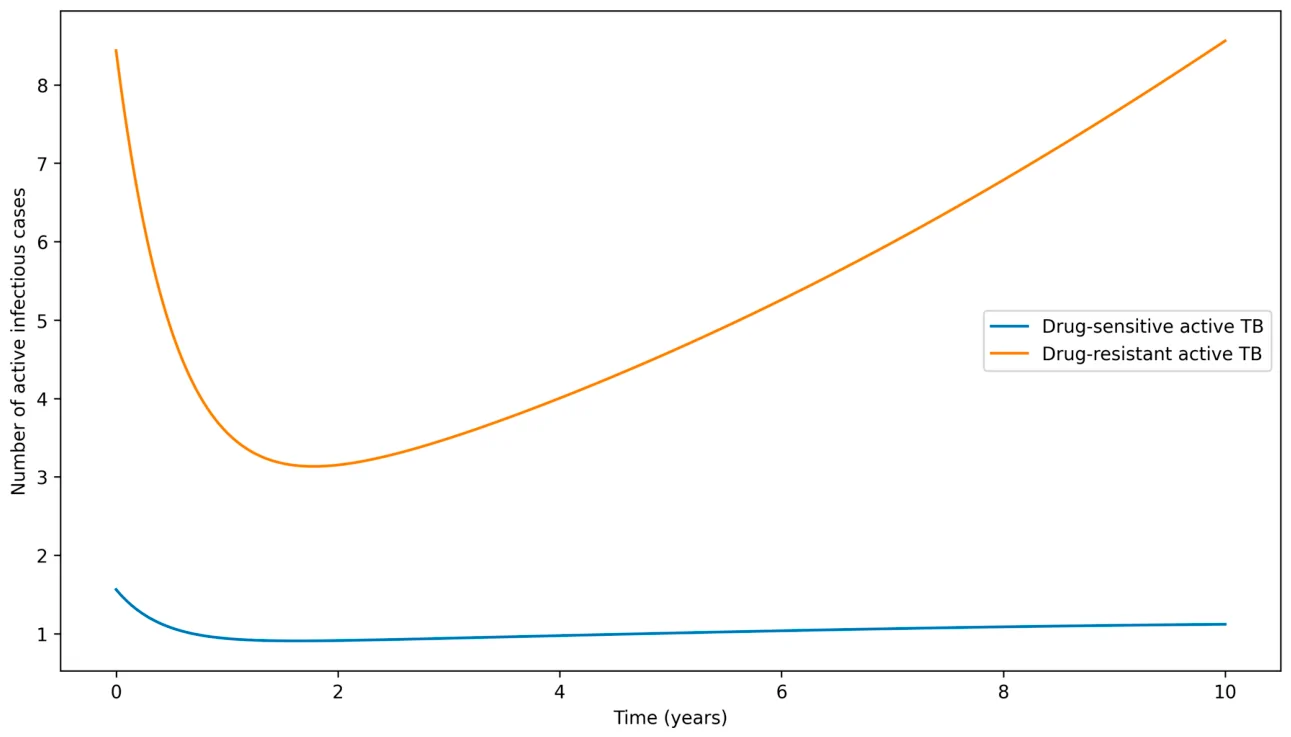
Simulated trajectories of drug-sensitive and drug-resistant tuberculosis under baseline assumptions. Ten-year modelled trajectories of active DS-TB and DR-TB cases generated using the conceptual two-strain compartmental model. The projections are illustrative scenario outputs and not epidemiological forecasts.

Ten-year simulations illustrated differences in projected DS-TB and DR-TB trajectories under the baseline assumptions (Figure 7). Both trajectories initially declined, while DR-TB subsequently persisted to a greater extent within the model. These trajectories illustrate mathematical behavior under the specified parameter assumptions and should not be interpreted as forecasts of the TB epidemic in O.R. Tambo District.

### 3.9. Community-Informed Scenario Outputs

**Figure 8:**
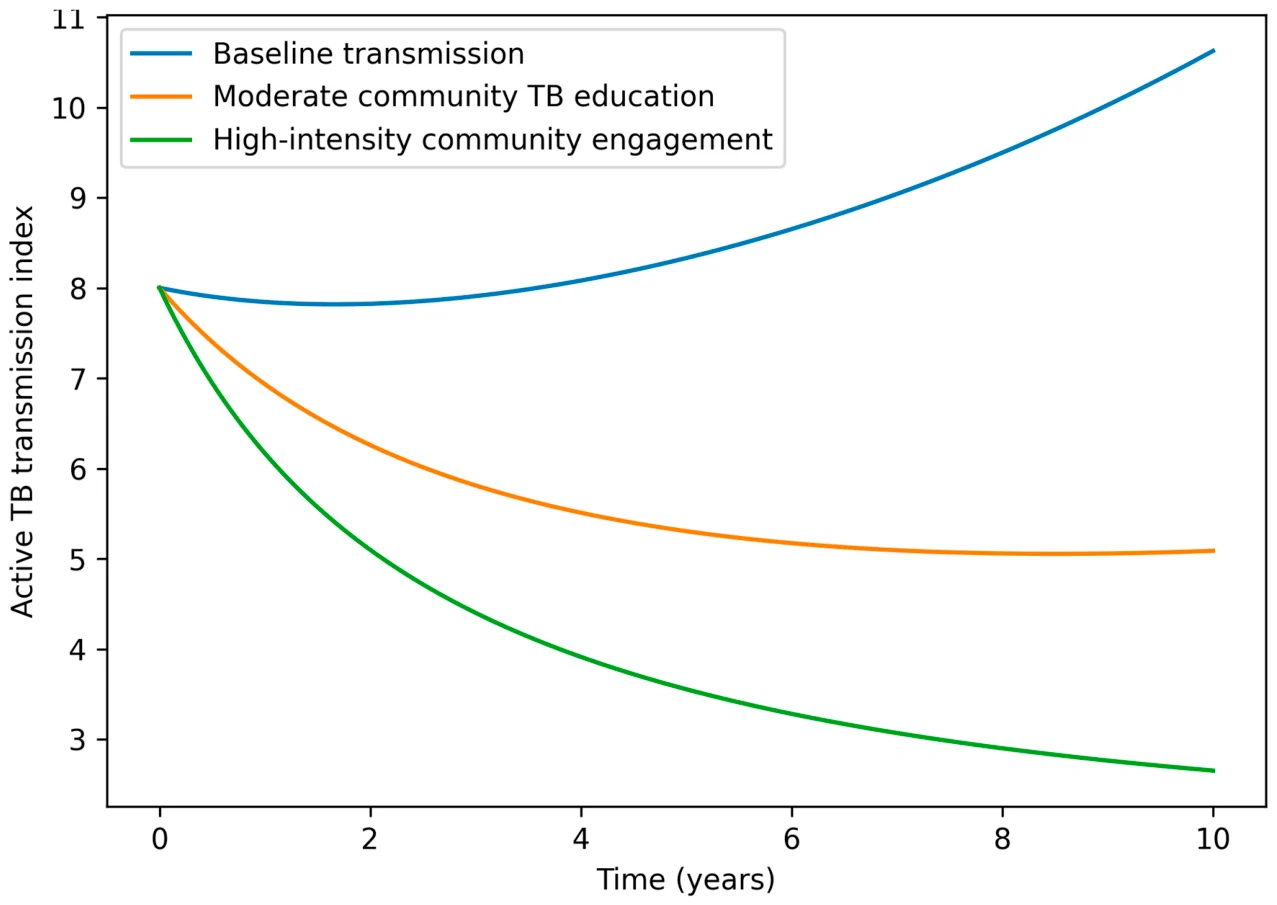
Simulated effects of community-informed scenarios on drug-sensitive and drug-resistant tuberculosis. Ten-year modelled DS-TB and DR-TB trajectories under baseline, moderate, and high-intensity community-informed scenarios. Intervention scenarios modify transmission, treatment initiation, and resistance amplification parameters. Outputs are exploratory projections under predefined assumptions and do not represent empirically observed intervention effects.

Community-informed scenarios explored hypothetical changes in three model parameters: transmission (β), treatment initiation (γ), and resistance amplification (α). Reductions in β represented improved infection-prevention practices; increases in γ represented earlier healthcare seeking and treatment initiation; and reductions in α represented improved treatment adherence and continuity. These parameter changes were predefined modelling assumptions rather than empirically measured effects of community engagement. Under a scenario assuming a 25% reduction in transmission coefficients, the illustrative R_0_ decreased from approximately 1.29 to 0.97 for DS-TB and from 2.04 to 1.53 for DR-TB. Thus, DS-TB fell below the theoretical transmission threshold of R_0_ = 1 in this scenario, whereas DR-TB remained above it. Across the predefined community-informed scenarios, the model generated approximately 40–60% reductions in projected DS-TB transmission and 20–35% reductions in projected DR-TB transmission relative to baseline. Higher-intensity parameter modifications produced larger reductions in projected active infections and slowed the expansion of DR-TB over the 10-year simulation period (Figure 8). These differences represent the mathematical consequences of the assumed parameter modifications and should not be interpreted as observed or causal effects of CBPR or community health-literacy interventions.

## 4. Discussion

This study integrated whole-genome sequencing (WGS), exploratory mathematical transmission modelling, and Community-Based Participatory Research (CBPR)-informed scenarios to examine drug-resistant tuberculosis (DR-TB) in a rural South African setting. The genomic analysis demonstrated a high frequency of resistance-associated mutations, with MDR-TB representing the largest resistance category. Lineages 2 and 4 predominated, and canonical resistance-associated mutations were identified in *rpoB*, *katG*, the *fabG1/inhA* promoter region, *gyrA*, and *rrs*. These findings are consistent with established molecular mechanisms of anti-tuberculosis drug resistance and the recognised contribution of Lineages 2 and 4 to the global DR-TB burden [29,30,31,32,33,34,35]. Although recurrent combinations of resistance-associated mutations were observed, these findings should not be interpreted as evidence of direct transmission. The study lacked pairwise SNP-based transmission thresholds and detailed epidemiological linkage data, including contact tracing, temporal relationships, and geographical information. The genomic findings, therefore, demonstrate genetic relatedness and recurring resistance signatures rather than confirmed transmission networks. Integration of genomic findings with epidemiological, temporal, and spatial information is required to strengthen inference of transmission [36,37,38]. The predominance of Lineages 2 and 4 is epidemiologically relevant. Lineage 2, including Beijing-family strains, has previously been associated with drug resistance and successful dissemination [30,31]. More complex resistance profiles appeared frequently among Lineage 2 isolates in the present dataset. However, the relatively small number of isolates precludes firm conclusions regarding lineage-specific associations with advanced drug resistance. These observations should therefore be regarded as descriptive and hypothesis-generating.

### 4.1. Interpretation of the Transmission Model

The two-strain model provided a conceptual framework for examining how DS-TB and DR-TB may respond differently to changes in transmission, treatment initiation, and resistance amplification. Under the selected baseline assumptions, illustrative R_0_ values were approximately 1.29 for DS-TB and 2.04 for DR-TB. When a 25% reduction in transmission coefficients was imposed, the corresponding scenario values decreased to approximately 0.97 and 1.53. These results demonstrate the mathematical consequences of the selected parameter assumptions rather than population-level estimates of transmission. Community-informed scenarios generated approximately 40–60% reductions in projected DS-TB transmission and 20–35% reductions in DR-TB transmission relative to baseline. These reductions should not be interpreted as evidence of intervention effectiveness. The model was not calibrated against longitudinal epidemiological data, and parameter modifications were predefined assumptions rather than empirically measured effects. The simulations instead identify plausible mechanisms and generate hypotheses for subsequent empirical investigation. WGS contributed biological context to the model by demonstrating the coexistence of susceptible and resistant profiles and identifying resistance-associated mutations. However, genomic frequencies were not used as numerical estimates of transmission, progression, treatment, or resistance-amplification parameters. This distinction prevents mutation prevalence or genomic relatedness from being interpreted as direct measures of epidemiological transmission.

### 4.2. Community-Informed Pathways and the Biosocial Framework

TB transmission is shaped by biological processes alongside behavioural, social, structural, and health-system conditions. CBPR was therefore incorporated as a conceptual framework to represent plausible community-level pathways that affect epidemiologically relevant processes. Rather than treating CBPR as a directly measurable model parameter, community-informed mechanisms were represented through hypothetical changes in transmission coefficients (β), treatment-initiation rates (γ), and resistance amplification (α). Within this framework, improved infection-prevention practices and health literacy may reduce transmission; earlier recognition of symptoms and healthcare-seeking may increase treatment initiation and shorten infectious periods; and improved treatment adherence and continuity may reduce opportunities for the amplification of resistance. These relationships are conceptually consistent with participatory approaches that seek to strengthen trust, identify barriers to care, and develop contextually appropriate interventions [38,39,40,41,42]. Importantly, CBPR was not prospectively implemented or evaluated as an intervention in this study. Consequently, the model does not demonstrate that community engagement causes the projected reductions in TB transmission. Instead, it provides a structured pathway to examine how measurable community- and health-system mechanisms could be incorporated into future transmission studies. Prospective studies should quantify these mechanisms using community engagement indicators, healthcare utilization measures, treatment outcomes, and longitudinal epidemiological data. The integration of WGS, modelling, and community-informed mechanisms therefore provides a biosocial framework rather than a validated intervention model. WGS characterizes pathogen resistance and diversity; mathematical modelling explores potential population-level dynamics; and community-informed scenarios represent social and behavioral mechanisms that conventional biomedical models may not capture.

### 4.3. Policy and Public Health Implications

The findings support strengthening genomic surveillance while ensuring that WGS is integrated with conventional epidemiological investigation. Genomic similarity alone should not determine public health action; contact tracing, temporal and spatial information, clinical histories, and surveillance data remain necessary for interpreting potential transmission patterns. The modelling component further suggests that interventions that act simultaneously on transmission, timely treatment initiation, and treatment continuity warrant prospective evaluation. Community engagement may provide one mechanism through which these processes can be addressed, particularly where stigma, delayed healthcare seeking, treatment interruption, and structural barriers limit TB control. However, the present scenario outputs should be considered hypothesis-generating rather than quantitative evidence of CBPR effectiveness.

## 5. Study Limitations

Several limitations should be considered when interpreting the findings. First, the genomic analysis included only 32 isolates and may not represent the full diversity of *M. tuberculosis* circulating in the O.R. Tambo District. Culture availability, specimen selection, sequencing success, and laboratory referral processes may also have introduced selection bias. Second, the cross-sectional WGS dataset and absence of pairwise SNP-based transmission analysis, detailed contact tracing, geographical linkage, and longitudinal sampling prevented reconstruction of transmission chains or differentiation between recent transmission and reactivation. Genomic similarities should therefore be interpreted as genetic relatedness rather than confirmed transmission.

Third, genomic data informed the biological structure of the mathematical model but did not provide estimates of its dynamic epidemiological parameters. The model was conceptual, literature-informed, and neither formally calibrated nor externally validated, nor subjected to probabilistic sensitivity analysis. Accordingly, the reported R_0_ values and scenario-related reductions are conditional model outputs, and confidence or credible intervals were not calculated. Fourth, the model simplified TB epidemiology by excluding factors such as HIV co-infection, reinfection, migration, age structure, stochastic variation, and tuberculosis preventive treatment. These omissions limit its ability to reproduce the epidemiological complexity of TB in the study setting. Finally, CBPR-informed parameter modifications were hypothetical rather than empirically estimated from a prospectively implemented community intervention. The projected effects, therefore, cannot establish the effectiveness or causal impact of CBPR. Larger prospective studies integrating longitudinal genomic, epidemiological, clinical, spatial, and community-level data are required to calibrate and validate the framework and quantify uncertainty. The genomic sample may be subject to selection bias because isolates were selected retrospectively based on culture availability, viability, DNA quality, and sequencing success. The high proportion of MDR-TB among sequenced isolates (46.9%) therefore cannot be considered representative of all notified TB cases in O.R. Tambo District. Population-level routine surveillance data indicates substantially lower proportions of RR-TB than those observed in this selected genomic sample. Accordingly, resistance proportions reported in this study should be interpreted as characteristics of the analysed isolate collection rather than estimates of district-level DR-TB prevalence.

## 6. Recommendations

Future research should expand WGS surveillance by using larger, more representative collections of isolates and integrating genomic data with contact tracing, spatial information, treatment histories, and longitudinal surveillance. This would permit a more robust assessment of genomic relatedness and potential transmission pathways. Mathematical models should be calibrated and validated using population-specific epidemiological data, accompanied by formal sensitivity and uncertainty analyses. Important determinants such as HIV co-infection, tuberculosis preventive treatment, reinfection, migration, and population heterogeneity should also be incorporated where appropriate. Prospective implementation research should define measurable indicators of community engagement and evaluate whether community-informed interventions affect healthcare-seeking, treatment initiation, adherence, infection prevention, and treatment outcomes. Such studies would enable the conceptual relationships proposed in this study to be empirically tested.

## 7. Conclusions

This study demonstrates the potential value of integrating WGS, mathematical transmission modelling, and community-informed mechanisms within a conceptual biosocial framework for DR-TB research. WGS characterized resistance-associated mutations, phylogenetic lineages, and genomic relatedness, while the two-strain model provided an exploratory representation of DS-TB and DR-TB dynamics. The modelling results are conditional on predefined assumptions and should not be interpreted as calibrated epidemiological estimates or evidence of CBPR effectiveness. Similarly, genomic relatedness does not establish direct transmission. CBPR was incorporated as a conceptual pathway through which behavioral, social, and health-system mechanisms could potentially influence transmission, treatment initiation, and resistance amplification. Future prospective studies combining larger longitudinal genomic datasets, epidemiological linkage information, calibrated transmission models, and empirically measured community-engagement indicators are required to validate the framework and determine its value for TB prevention and control in high-burden settings.

## Figures and Tables

**Table 1:** Parameter values used in deterministic two-strain (drug-sensitive and drug-resistant) tuberculosis transmission model.

Parameter (Symbol)	Definition	Units	Baseline	Moderate Community Education ^a^	High-Intensity Community Engagement ^b^	Source/Justification
*β_s_*	Transmission rate (drug-sensitive TB)	year^−1^	0.44	0.33	0.22	Calibrated to give *R*_0*s*_ ≈ 1.29 [16,17,18]
*β_r_*	Transmission rate (drug-resistant TB)	year^−1^	1.10	0.83	0.55	Calibrated to give *R*_0*r*_ ≈ 2.04 [16,17,18]
*σ_s_*	Progression rate from latent to active disease (DS)	year^−1^	0.10	0.10	0.10	The literature [19,20]
*σ_r_*	Progression rate from latent to active disease (DR)	year^−1^	0.10	0.10	0.10	Assumed same as DS [19]
*γ_s_*	Treatment initiation rate (DS)	year^−1^	6.50	8.13	9.75	Inverse of average diagnostic delay [21,22]
*γ_r_*	Treatment initiation rate (DR)	year^−1^	4.00	5.00	6.00	Longer delays for DR-TB [21,22]
*ρ_s_*	Successful treatment recovery rate (DS)	year^−1^	0.85	0.85	0.85	Typical treatment success [23,24]
*ρ_r_*	Successful treatment recovery rate (DR)	year^−1^	0.60	0.60	0.60	Lower success for DR-TB [23,24]
*α*	Resistance amplification rate (DS → DR during treatment)	year^−1^	0.10	0.05	0.02	Assumed reduction with improved adherence [25,26]
*μ*	Background (natural) mortality rate	year^−1^	0.011	0.011	0.011	South African life expectancy (≈67 years) [27]
*d_s_*	TB-related mortality rate (DS)	year^−1^	0.05	0.05	0.05	The literature [28,29]
*d_r_*	TB-related mortality rate (DR)	year^−1^	0.10	0.10	0.10	Higher mortality for DR-TB [28,29]
*N*	Total population size (modelled)	persons	100,000	100,000	100,000	Hypothetical closed population for simulations
Time horizon	Simulation period	years	10	10	10	As specified in Methods
Derived quantities						
*R*_0*s*_	Basic reproduction number (DS-TB) ^c^	–	1.29	0.97	0.65	Calculated from model parameters
*R*_0*r*_	Basic reproduction number (DR-TB) ^c^	–	2.04	1.53	1.02	Calculated from model parameters

**^a^** Moderate community education scenario: 25% reduction in transmission rates (*β_s_* and *β_r_*), 25% increase in treatment initiation rates (*γ_s_* and *γ_r_*), and 50% reduction in resistance amplification (*α*). ^b^ High-intensity community engagement scenario: 50% reduction in transmission rates (*β_s_* and *β_r_*), 50% increase in treatment initiation rates (*γ_s_* and *γ_r_*), and 80% reduction in resistance amplification (*α*). **^c^** *R*_0_ calculated using the next-generation matrix for the infectious compartments, with the simplified relationship *R*_0_ ≈ *β*/(*γ* + *μ* + *d*).

**Table 2:** Evidence Base and Justification for Epidemiological Parameters Used in the DS–DR Tuberculosis Transmission Model.

Parameter	Link to Table 1	Evidence Base and Justification	Reference(s)
DS-TB transmission rate (*β_s_*)	Baseline: 0.44 year^−1^; modified under intervention scenarios	Model-calibrated transmission parameter used to represent effective transmission of DS-TB. Published TB transmission and mathematical modelling studies informed the biological structure and plausible transmission dynamics. The exact value was not estimated from the WGS dataset.	[16,17,18]
DR-TB transmission rate (*β_r_*)	Baseline: 1.10 year^−1^; modified under intervention scenarios	Model-calibrated parameter representing effective transmission of DR-TB. It was specified for comparative scenario analysis and was not directly estimated from genomic clustering, mutation frequencies, or the 32 WGS isolates.	[16,17,18]
DS-TB progression rate (*σ_s_*)	0.10 year^−1^	Assumed latent-to-active disease progression rate informed by published evidence on the natural history and timing of progression to active TB.	[19,20]
DR-TB progression rate (*σ_r_*)	0.10 year^−1^	Assumed equal to the DS-TB progression rate because the present genomic dataset did not provide an empirical estimate of differential progression according to resistance phenotype.	[19,20]
DS-TB treatment-initiation rate (*γ_s_*)	Baseline: 6.50 year^−1^; increased in intervention scenarios	Model parameter representing movement from infectious DS-TB to treatment. Intervention-related increases represent earlier symptom recognition, healthcare seeking, diagnosis and treatment initiation. The exact rate is a modelling assumption rather than an estimate from the WGS data.	[22]
DR-TB treatment-initiation rate (*γ_r_*)	Baseline: 4.00 year^−1^; increased in intervention scenarios	A lower baseline rate was used to represent potentially longer pathways to appropriate DR-TB treatment. South African evidence demonstrates clinically important delays between diagnosis and initiation of DR-TB treatment.	[21,22,23]
DS-TB treatment recovery rate (*ρ_s_*)	0.85 year^−1^	Informed by the comparatively high treatment success reported for drug-susceptible TB. The parameter is used as a model transition rate and should not be interpreted as identical to the reported proportion of patients with successful treatment outcomes.	[22]
DR-TB treatment recovery rate (*ρ_r_*)	0.60 year^−1^	A lower recovery rate was specified to reflect poorer treatment outcomes and greater therapeutic complexity associated with MDR/RR-TB compared with drug-susceptible TB.	[22,23,24]
Resistance amplification (*α*)	Baseline: 0.10 year^−1^; 0.05 moderate; 0.02 high-intensity	Represents amplification of drug resistance during DS-TB treatment. The baseline value is a modelling assumption. Reductions in intervention scenarios represent the expected direction of effect from improved treatment adherence, retention in care and continuity of treatment rather than empirically measured intervention effects.	[25,26]
Background mortality (*μ*)	0.011 year^−1^	Approximate demographic mortality parameter used for the hypothetical model population and informed by South African demographic estimates.	[27]
DS-TB excess mortality (*d_s_*)	0.05 year^−1^	Assumed TB-specific excess mortality parameter informed by evidence on the natural history, mortality and outcomes of TB. The precise numerical rate was not estimated from the study dataset.	[19,22]
DR-TB excess mortality (*d_r_*)	0.10 year^−1^	A higher excess mortality parameter was assumed for DR-TB to represent its poorer outcomes and greater treatment complexity relative to DS-TB.	[22,23,24,28]
Population (*N*)	100,000 persons	Hypothetical closed model population selected to facilitate comparison of transmission trajectories across scenarios; not the observed population size of the study area.	Model assumption
Simulation horizon	10 years	Predefined period used to examine medium-term differences between baseline, moderate community education and high-intensity community engagement scenarios.	

Note: Table 2 should be interpreted together with Table 1. Table 1 reports the numerical parameter values used in the model and their modification across the three scenarios, whereas Table 2 distinguishes parameters informed by published evidence from values introduced as explicit modelling assumptions. Literature citations support the epidemiological rationale, direction and, where applicable, plausible magnitude of the parameters; they should not be interpreted as direct empirical sources for every exact numerical value used in Table 1.

## Data Availability

The data supporting the findings of this study are available from the corresponding author upon reasonable request, subject to applicable ethical and institutional requirements.

## Abbreviations

AF	Allele Frequency
AIDS	Acquired Immunodeficiency Syndrome
AMPure XP	Agencourt AMPure XP Magnetic Beads
BWA	Burrows–Wheeler Aligner
CAS	Central Asian Strain
CBPR	Community-Based Participatory Research
DNA	Deoxyribonucleic Acid
DR-TB	Drug-Resistant Tuberculosis
DS-TB	Drug-Sensitive Tuberculosis
DOTS	Directly Observed Treatment, Short-course
GATK	Genome Analysis Toolkit
HIV	Human Immunodeficiency Virus
H37Rv	*Mycobacterium tuberculosis* Reference Strain H37Rv
LTBI	Latent Tuberculosis Infection
MDR-TB	Multidrug-Resistant Tuberculosis
Mtb	*Mycobacterium tuberculosis*
PCR	Polymerase Chain Reaction
pre-XDR-TB	Pre-Extensively Drug-Resistant Tuberculosis
RR-TB	Rifampicin-Resistant Tuberculosis
SNP	Single-Nucleotide Polymorphism
TB	Tuberculosis
TDR-TB	Totally Drug-Resistant Tuberculosis
USAP	Unified Sequence Analysis Pipeline
WGS	Whole-Genome Sequencing
WHO	World Health Organization
XDR-TB	Extensively Drug-Resistant Tuberculosis

## References

[1] Farnia P., Velayati A.A., Ghanavi J., Farnia P. (2025). Tuberculosis: An Ongoing Global Threat. Proteins in Mycobacterium Tuberculosis: Functions and Therapeutic Advances.

[2] Dheda K., Mirzayev F., Cirillo D.M., Udwadia Z., Dooley K.E., Chang K.C., Omar S.V., Reuter A., Perumal T., Horsburgh C.R. (2024). Multidrug-Resistant Tuberculosis. Nat. Rev. Dis. Primers.

[3] Said H., Ratabane J., Erasmus L., Gardee Y., Omar S., Dreyer A., Ismail F., Bhyat Z., Lebaka T., van der Meulen M. (2021). Distribution and Clonality of Drug-Resistant Tuberculosis in South Africa. BMC Microbiol..

[4] Faye L.M., Hosu M.C., Apalata T. (2024). Drug-Resistant Tuberculosis in Rural Eastern Cape, South Africa: A Study of Patients’ Characteristics in Selected Healthcare Facilities. Int. J. Environ. Res. Public Health.

[5] Al-Jardani A., Al Yaquobi F., Adikaram C., Al Wahaibi A., Al-Balushi L., Al-Zadjali S., Al Abri B., Al-Thuhli K., Al-Abri S. (2023). Genomic and Geospatial Epidemiology of Mycobacterium tuberculosis in Oman: First National Insight Using Whole Genome Sequencing. Int. J. Infect. Dis..

[6] Coleman M., Martinez L., Theron G., Wood R., Marais B. (2022). Mycobacterium tuberculosis Transmission in High-Incidence Settings—New Paradigms and Insights. Pathogens.

[7] Naidoo K., Perumal R., Cox H., Mathema B., Loveday M., Ismail N., Omar S.V., Georghiou S.B., Daftary A., O’Donnell M. (2024). The Epidemiology, Transmission, Diagnosis, and Management of Drug-Resistant Tuberculosis—Lessons from the South African Experience. Lancet Infect. Dis..

[8] Rodríguez-Grande C., Vallejo-Godoy S., Martínez-Lirola M., Saleeb S.M., Herranz M., Buenestado-Serrano S., Marcos-Abellán A., Barroso-García P., Nieto S.R., Escabias-Machuca F. (2026). Long-Term Refined Genomic Analysis of Tuberculosis Clusters to Distinguish between Ongoing Transmission, Reactivations or Diagnostic Delays, Almería, Spain, 2003 to 2024. Eurosurveillance.

[9] Peter O.J., Abidemi A., Fatmawati F., Ojo M.M., Oguntolu F.A. (2024). Optimizing Tuberculosis Control: A Comprehensive Simulation of Integrated Interventions Using a Mathematical Model. Math. Model. Numer. Simul. Appl..

[10] Nasrin S., Chowdhury N.M. (2021). Dealing with Tuberculosis: Factors of Tuberculosis Medication Adherence among Marginalized Communities: A Scoping Literature Review. Int. J. Community Med. Public Health.

[11] Khumalo L., Ellis M., Limson J. (2026). A Relational Lens to Community-Based Participatory Research: An Analysis of Case Studies of Engaged Research in South Africa. S. Afr. J. Sci..

[12] Department of Cooperative Governance and Traditional Affairs (CoGTA) (2020). District Development Model Profile: O.R. Tambo District Municipality.

[13] Municipalities of South Africa (2026). O.R. Tambo District Municipality (DC15): Map and Overview. https://municipalities.co.za/overview/106/or-tambo-district-municipality.

[14] Statistics South Africa (2022). Census 2022 Municipal Profile: Eastern Cape.

[15] Eastern Cape Department of Social Development (2024). O.R. Tambo District Annual Performance Plan 2024/2025.

[16] Knight G.M., Dodd P.J., Grant A.D., Fielding K.L., Churchyard G.J., White R.G. (2015). Tuberculosis prevention in South Africa. PLoS ONE.

[17] Houben R.M.G.J., Dodd P.J. (2016). The global burden of latent tuberculosis infection: A re-estimation using mathematical modelling. PLoS Med..

[18] Dodd P.J., Yuen C.M., Becerra M.C., Revill P., Jenkins H.E., Seddon J.A. (2018). Potential effect of household contact management on childhood tuberculosis: A mathematical modelling study. Lancet Glob. Health.

[19] Tiemersma E.W., van der Werf M.J., Borgdorff M.W., Williams B.G., Nagelkerke N.J.D. (2011). Natural history of tuberculosis: Duration and fatality of untreated pulmonary tuberculosis in HIV negative patients: A systematic review. PLoS ONE.

[20] Behr M.A., Edelstein P.H., Ramakrishnan L. (2018). Revisiting the timetable of tuberculosis. BMJ.

[21] Cox H., Dickson-Hall L., Ndjeka N., Van’t Hoog A., Grant A., Cobelens F., Stevens W., Nicol M. (2017). Delays and loss to follow-up be-fore treatment of drug-resistant tuberculosis following implementation of Xpert MTB/RIF in South Africa: A retrospective cohort study. PLoS Med..

[22] World Health Organization (2022). Global Tuberculosis Report 2022.

[23] World Health Organization (2022). WHO Consolidated Guidelines on Tuberculosis. Module 4: Treatment—Drug-Resistant Tuberculosis Treatment, 2022 Update.

[24] Mirzayev F., Viney K., Linh N.N., Gonzalez-Angulo L., Gegia M., Jaramillo E., Zignol M., Kasaeva T. (2020). World Health Organization recommendations on the treatment of drug-resistant tuberculosis, 2020 update. Eur. Respir. J..

[25] Alipanah N., Jarlsberg L., Miller C., Linh N.N., Falzon D., Jaramillo E., Nahid P. (2018). Adherence interventions and outcomes of tuberculosis treatment: A systematic review and meta-analysis of trials and observational studies. PLoS Med..

[26] Law S., Daftary A., O’Donnell M., Padayatchi N., Calzavara L., Menzies D. (2019). Interventions to improve retention-in-care and treatment adherence among patients with drug-resistant tuberculosis: A systematic review. Eur. Respir. J..

[27] Statistics South Africa (2022). Mid-Year Population Estimates.

[28] Falzon D., Schünemann H.J., Zignol M., Mirzayev F., Kasaeva T. (2020). WHO guidance on multidrug-resistant tuberculosis treatment: Using and communicating the evidence. Eur. Respir. J..

[29] Dekhil N., Mardassi H. (2023). Genomic Changes Underpinning the Emergence of a Successful Mycobacterium tu-berculosis Latin American and Mediterranean Clonal Complex. Front. Microbiol..

[30] Atavliyeva S., Auganova D., Tarlykov P. (2024). Genetic Diversity, Evolution and Drug Resistance of Mycobacterium tuberculosis Lineage 2. Front. Microbiol..

[31] Klopper M., Heupink T.H., Hill-Cawthorne G., Streicher E.M., Dippenaar A., De Vos M., Abdallah A.M., Limberis J., Merker M., Burns S. (2020). A Landscape of Genomic Alterations at the Root of a Near- Untreatable Tuberculosis Epidemic. BMC Med..

[32] Faye L.M., Hosu M.C., Vasaikar S., Dippenaar A., Oostvogels S., Warren R.M., Apalata T. (2023). Spatial Distribution of Drug-Resistant Mycobacterium tuberculosis Infections in Rural Eastern Cape Province of South Africa. Pathogens.

[33] Conceição E.C., Loubser J., Guimarães A.E.D.S., Sharma A., Rutaihwa L.K., Dippenaar A., Salvato R.S., de Paula Souza e Guimarães R.J., da Silva Lourenço M.C., Barros W.A. (2024). A Genome-Focused Investigation Reveals the Emergence of a Mycobacterium tuberculosis Strain Related to Multidrug-Resistant Tuberculosis in the Amazon Region of Brazil. Microorganisms.

[34] Chizimu J.Y., Solo E.S., Bwalya P., Tanomsridachchai W., Chambaro H., Shawa M., Kapalamula T.F., Lungu P., Fukushima Y., Mukonka V. (2021). Whole-Genome Sequencing Reveals Recent Transmission of Multi-drug-Resistant Mycobacterium tuberculosis CAS1-Kili Strains in Lusaka, Zambia. Antibiotics.

[35] World Health Organization (WHO) (2023). Global Tuberculosis Report 2023.

[36] Lan Y., Rancu I., Chitwood M.H., Sobkowiak B., Nyhan K., Lin H.H., Wu C.Y., Mathema B., Brown T.S., Colijn C. (2025). Integrating Genomic and Spatial Analyses to Describe Tuberculosis Transmission: A Scoping Review. Lancet Microbe.

[37] Smith J.P., Cohen T., Dowdy D., Shrestha S., Gandhi N.R., Hill A.N. (2023). Quantifying Mycobacterium tuberculosis Transmission Dynamics across Global Settings: A Systematic Analysis. Am. J. Epidemiol..

[38] Chavez-Rimache L., Ugarte-Gil C., Brunette M.J. (2023). The Community as an Active Part in the Implementation of Interventions to Prevent and Control Tuberculosis: A Scoping Review. medRxiv.

[39] Lazarus S., Naidoo A.V., May B., Williams L., Demas G., Filander F.J. (2014). Lessons Learned from a Community-Based Participatory Research Project in a South African Rural Context. S. Afr. J. Psychol..

[40] Riccardi M.T., Pettinicchio V., Di Pumpo M., Altamura G., Nurchis M.C., Markovic R., Šagrić Č., Stojanović M., Rosi L., Damiani G. (2023). Community-Based Participatory Research to Engage Disadvantaged Communities: Levels of Engagement Reached and How to Increase It: A Systematic Review. Health Policy.

[41] Moore T.R., Chusan Y.A., Sanderson E., Calancie L., Hennessy E., Appel J.M., Ulseth M., Economos C.D. (2025). Community-Led Change: Progress toward Policy, Systems, and Environmental Impacts through the Catalyzing Communities Initiative. PLoS ONE.

[42] Hayward S.E., Vanqa N., Makanda G., Tisile P., Ngwatyu L., Foster I., Mcinziba A.A., Biewer A., Mbuyamba R., Galloway M. (2024). “As a Patient I Do Not Belong to the Clinic, I Belong to the Community”: Co-Developing Multi-Level, Person-Centered Tuberculosis Stigma Interventions in Cape Town, South Africa. BMC Glob. Public Health.

